# P-443. Outcomes in Pneumocystis Pneumonia Hospitalizations in Patients with HIV: A County Hospital Study 2015-2022

**DOI:** 10.1093/ofid/ofae631.643

**Published:** 2025-01-29

**Authors:** Erica Chow, Kirk B Fetters, James C Ziegenbein, Timothy Hatlen

**Affiliations:** Harbor-UCLA, Los Angeles, California; Harbor-UCLA Medical Center, Denver, Colorado; Harbor UCLA, Torrance, California; Harbor-UCLA Medical Center, Denver, Colorado

## Abstract

**Background:**

Therapy related complications, morbidity and mortality are still problematic in treating pneumocystis jirovecii pneumonia (PJP) in persons living with HIV (PWH) on antiretroviral therapy (ART). Our study aims to outline patient demographics and hospital outcomes among PWH in a safety-net hospital during the contemporary ART era.

**Figure:**
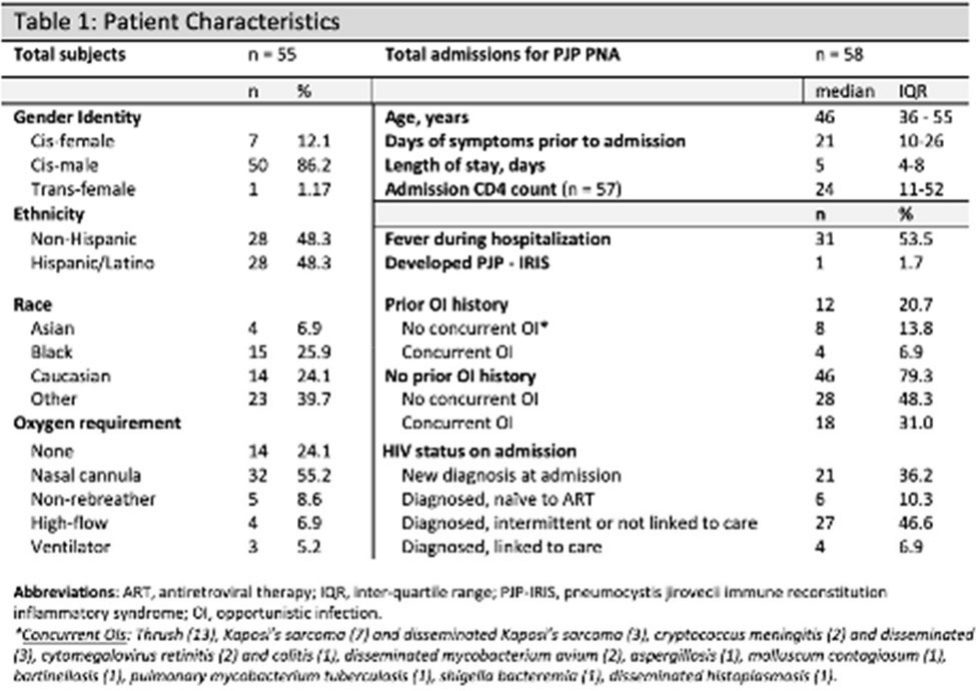

**Methods:**

We retrospectively reviewed and extracted data from inpatient encounters with HIV and PJP between 2015-2022 at a safety-net hospital in Los Angeles County, California

**Figure:**
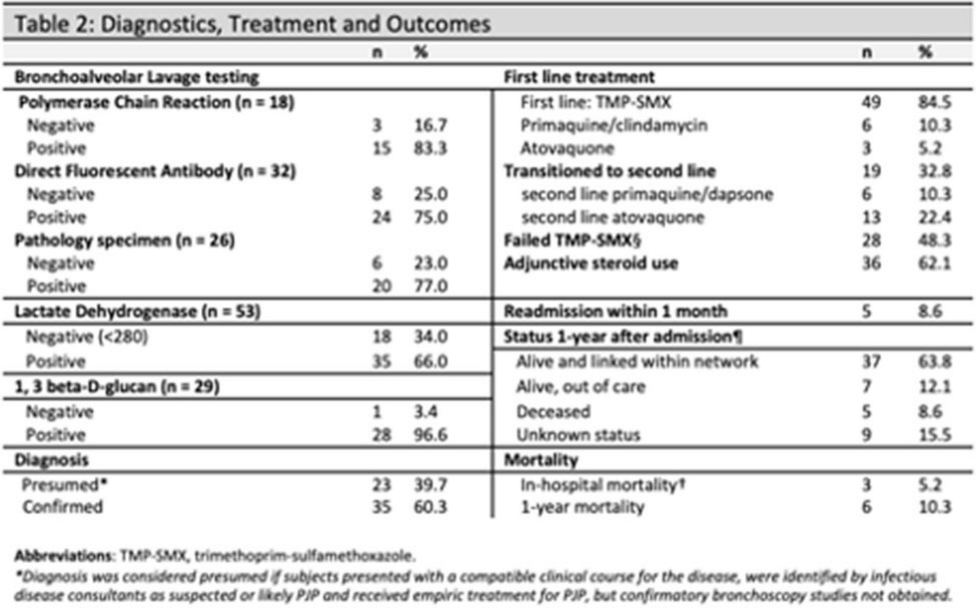

**Results:**

We identified 55 PWH (58 admissions) with confirmed or presumed PJP. Only 6.9% had a known HIV diagnosis and linked to care the prior year. 37.9% had concurrent opportunistic infections (OIs), and 20.7% had prior OI history. Treatment with trimethoprim-sulfamethoxazole (TMP-SMX) was used in 84.5% of cases in whom 48% had treatment related adverse effects. 62.1% received steroids. At one-year post-hospitalization, 63.8% were linked to care and 27.6% were lost to follow up or out of network. Inpatient mortality was 5.2%, and 10.3% died at 1-year. Higher oxygen requirements predicted a higher 1-year mortality (OR 2.68, 95%CI = 1.28 – 5.64, p = < 0.01), as did necessity of TMP-SMX alternatives (OR 18.8, p = 0.003, 95% CI 2.72 – 129.73).

**Conclusion:**

A total of 32.8% of patients failed TMP-SMX. These patients also had higher 12-month mortality rate, likely reflecting underlying host comorbidities and disease severity. Atovaquone is inferior to TMP-SMX and primaquine-clindamycin is non-inferior to TMP-SMX, however, only in mild-moderate disease. There is a paucity of data for alternative treatments for severe disease.

Concurrent OI were found in 37.9%, of whom 81.8% had no prior history of OI. Of the three inpatient mortalities, two had concurrent OIs, emphasizing the clinical importance of screening for other OI in patients who present with PJP.

In-hospital mortality was 5.2%, compared to 9 -17% in other studies; 1-year mortality was 10.3% compared to estimates of 37-53% during early ART period, suggesting a positive trend compared to both pre- and early ART outcomes. The one-year follow-up mortality of 10.3% and having only 63.8% successfully linked with in-network HIV care underscores an opportunity to improve linkage and retention strategies for PWH.

**Disclosures:**

**All Authors**: No reported disclosures

